# Designing Microfluidic Devices to Sort Haematopoietic Stem Cells Based on Their Mechanical Properties

**DOI:** 10.1155/2019/8540706

**Published:** 2019-09-05

**Authors:** Mingming Du, Dean Kavanagh, Zhibing Zhang, Neena Kalia

**Affiliations:** ^1^School of Chemical Engineering, University of Birmingham, Birmingham, B15 2TT, UK; ^2^Institute of Cardiovascular Sciences, University of Birmingham, Birmingham, B15 2TT, UK

## Abstract

**Aim:**

Few haematopoietic stem cells (HSCs) injected systemically for therapeutic purposes actually reach sites of injury as the vast majority become entrapped within pulmonary capillaries. One promising approach to maintain circulating HSC numbers would be to separate subpopulations with smaller size and/or greater deformability from a heterogeneous population. This study tested whether this could be achieved using label-free microfluidic devices.

**Methods:**

2 straight (A-B) and 3 spiral (C-E) devices were fabricated with different dimensions. Cell sorting was performed at different flow rates after which cell diameter and stiffness were determined using micromanipulation. Cells isolated using the most efficient device were tested intravitally for their ability to home to the mouse injured gut.

**Results:**

Only straight Device B at a high flow rate separated HSCs with different mechanical properties. Side outlets collected mostly deformable cells (nominal rupture stress/*σ*_R_ = 6.81 kPa; coefficient of variation/CV = 0.31) at a throughput of 2.3 × 10^5^ cells/min. All spiral devices at high flow rates separated HSCs with different stiffness and size. Inner outlets collected mostly deformable cells in Devices C (*σ*_R_ = 25.06 kPa; CV = 0.26), D (*σ*_R_ = 22.21 kPa; CV = 0.41), and E (*σ*_R_ = 29.26 kPa; CV = 0.27) at throughputs of 2.3 × 10^5^ cells/min, 1.5 × 10^5^ cells/min, and 1.6 × 10^5^ cells/min, respectively. Since Device C separated cells with higher efficiency and throughput, it was utilized to test the homing ability of separated cells *in vivo*. Significantly more deformable cells were observed trafficking through the injured gut—interestingly, increased retention was not observed.

**Conclusion:**

This study applied microfluidics to separate subpopulations from one stem cell type based on their intrinsic mechanical heterogeneity. Fluid dynamics within curved devices most effectively separated HSCs. Such devices may benefit cellular therapy.

## 1. Introduction

Despite emerging evidence that exogenous haematopoietic stem/progenitor cells (HSCs) can help treat ischaemic and inflammatory disorders, their benefits are either minor or transient. The preferred delivery method for stem cells (SCs) is direct infusion into the bloodstream as it is simpler, less invasive, and less expensive than other routes [1, 2]. Systemic delivery relies on the microvessels of injured organs capturing HSCs as they home to and circulate through them. However, this delivery route is associated with poor HSC homing to injury sites and thus limited local retention. Indeed, most injected cells become entrapped in the small capillaries of off-target sites [3]. This event is most significant in the lungs, a phenomenon described as the “pulmonary first-pass effect,” and leaves few cells in the peripheral blood thereby reducing numbers available for capture [4–6]. There is little doubt that therapeutic efficacy is limited due to insufficient SC homing to target organs. To improve recruitment, several strategies have been employed including genetically modifying adhesion molecule expression on the SC surface or pretreating with inflammatory stimuli [7, 8]. Although adhesive interactions within local tissue microcirculation are indeed increased, these strategies do not improve circulating SC numbers and thus their actual homing to injury sites, a prerequisite to adhesion. This limits the effectiveness of such proadhesive strategies. Therefore, alternative or additional approaches are required to encourage successful passage of SCs through nonspecific sites and maintain an available pool of circulating cells.

Mechanical properties, namely, size and deformability, of circulating cells are important parameters affecting pulmonary entrapment [9]. Since the diameter of capillaries are smaller than the typical size of HSCs, they would need to undergo rapid deformation within a short time to successfully pass through. This is a particular problem for MSCs which are a larger cell type compared to HSCs [4, 10]. In addition to size, poor SC deformability may also contribute to their inability to pass through pulmonary capillaries. The importance of mechanical deformation in preventing cellular microvascular entrapment is highlighted by the fact that it is a key player in permitting neutrophils, with diameters of 6-8 *μ*m, to traverse smaller pulmonary capillaries, with diameters of 2-15 *μ*m [11, 12]. In their transit through pulmonary and systemic microcirculations, neutrophils undergo a significant deformation when subjected to mechanical stimulation in narrow capillaries [13]. Therefore, one promising approach to maintain circulating numbers would be to separate subpopulations of HSCs with a smaller size and/or less stiffness from a potentially heterogeneous population. This would theoretically avoid the “pulmonary first-pass” problem and ensure HSCs are retained in the circulation. High-throughput HSC sorting could be achieved using label-free techniques that make use of microfluidics and the intrinsic physical and mechanical properties of cells. Various microfluidic devices, typically with dimensions < 1 mm and Reynolds number (Re) < 100, are available which use different forces to separate cells migrating across distinct streamlines into equilibrium positions within the fluid. In straight channels, inertial lift forces are responsible for focusing cells of different size to different equilibrium positions [14–16]. Inertial microfluidics has attracted the most interest in the past two decades due to its precise manipulation, simple structure, and high throughput (10^6^ cells/min). Size-dependent or inertial lift forces can be combined with deformability-dependent forces for separation purposes when a curvature is added to a microfluidic device [17, 18]. A secondary flow arises due to the mismatch of velocity between fluid near the centerline and the side walls as it passes around a curve. The rotational Dean drag force is one such secondary flow type found in curved or spiral microchannel geometries that can be balanced with inertial forces to focus cells into a particular outlet channel of a separating device [17–19].

The use of microfluidic devices to isolate HSC subpopulations based on their physical and mechanical properties has not previously been investigated. Therefore, in the present study, the inertial lift forces in straight microchannels and the coupling of inertial and secondary Dean flows in spiral microchannels were used to sort HSCs. Five different microfluidic devices were designed in order to identify the most effective and efficient device geometry for separating HSCs into distinct subgroups differing in their mechanical stiffness. Two straight and three spiral devices were fabricated in which the channel aspect ratio (AR; width/height) and flow parameters were modified. To assess whether a separated subpopulation of more deformable HSCs resulted in improved homing and adhesion *in vivo*, fluorescent intravital microscopy (IVM) was used to monitor individual HSC trafficking to the injured mouse small intestine. This novel study demonstrates that not all HSCs have the same stiffness, that HSCs with more deformable mechanical properties can indeed be isolated with high throughput using spiral microchannels and, more importantly, these deformable cells home to injured tissues with greater efficacy.

## 2. Materials and Methods

### 2.1. Fabrication of Microfluidic Chips

Both rectangular straight microchannels, Devices A and B, had one inlet and three outlets (center and two sides) but had different geometries. They were used to investigate the effects of the cell aspect ratio and channel hydraulic diameter on cell sorting based on the simulation results of Kilimnik and colleagues [19]. All three rectangular spiral microchannels, Devices C, D, and E, also had one inlet but two outlets (inner and outer) and different geometries (Supplementary [Supplementary-material supplementary-material-1]; Tables [Supplementary-material supplementary-material-1] and [Supplementary-material supplementary-material-1]) and were used to investigate the effect of channel curvature on cell sorting following the work of Lee and colleagues [20]. Microchannels were fabricated using soft lithography techniques based on standard methods previously described [21, 22]. Briefly, a photolithography mask or template of the microchannel device was drawn in AutoCAD, a design software package, and produced on a silicon substrate mold. A polydimethylsiloxane (PDMS) slab was peeled from the mold and bonded with a flat PDMS substrate. Plastic tubing was inserted into the inlet port and connected to a syringe pump which injected cells and into outlet ports to collect cells (Supplementary [Supplementary-material supplementary-material-1]).

### 2.2. Culture of the HSC Cell Line: HPC-7s

Since HSCs are rare cells, it is difficult to isolate sufficient numbers for experimental research. Therefore, all studies utilized an immortalised murine haematopoietic progenitor cell line, namely, HPC-7 (a kind gift from Professor Leif Carlsson, Sweden). HPC-7 cells display many critical characteristics of primary HSCs, including high expression of common murine HSC markers as well as being lineage negative. Crucially, HPC-7s are able to fully reconstitute haematopoiesis when injected into a lethally irradiated host [23, 24]. In addition, we have demonstrated that HPC-7s express adhesion molecules known to be present on primary HSCs and have previously used HPC-7 to investigate their hepatic and intestinal homing [25, 26]. HSCs were maintained in StemPro-34 SFM supplemented with the manufacturer's media supplement (Invitrogen, UK), 100 ng/ml SCF (Invitrogen), L-glutamine (PAA, Somerset, UK), and penicillin and streptomycin (PAA). For sorting purposes, 4 ml HSC suspension at a density of 2 × 10^6^/ml was injected into each channel at various flow rates. Subpopulations harvested from the outlets were counted and subsequently processed for further mechanical studies.

### 2.3. Mechanical Analysis of Harvested Cells Using Micromanipulation

Harvested HSCs were mechanically tested with the micromanipulation technique, as previously described in detail [27, 28]. Both cell separation and mechanical measurements were performed at room temperature, with experiments completed within 2 hours from separation to measurement. Briefly, the technique involves compression of a single cell between the flat end of a borosilicate glass probe and the bottom of a glass chamber. Suspended single cells were allowed to settle to the bottom of the transparent chamber, and images were captured with a side-view high-speed digital camera. Cells settled immediately (due to gravity), and 20 cells per group were mechanically tested within 30 minutes. No change in stiffness was noted for the same cell population during the time they were in the medium. The probe, with a 25 *μ*m diameter flat end, was driven down by a stepping motor towards the single cells. The probe was connected to a force transducer (406A-ER, Aurora Scientific Inc., Canada) which simultaneously collected the data of instantaneous force imposed on single cells at a frequency of 50 Hz. From the measurement, force versus time and force versus displacement data could be obtained. Single cells were compressed to rupture between two parallel surfaces. The cell diameter, prior to applying force, was directly measured from its image on the TV monitor. Force versus displacement data up to cell rupture was obtained to determine the nominal rupture stress (NRS; *σ*_R_), which represented the mechanical strength of the cells. Higher NRS values indicated stiffer cells with low deformability and *vice versa*. Mean values of size, NRS, and the NRS coefficient of variation (CV) were calculated to assess separation efficiency. Initial experiments demonstrated that HSCs had a wide variation in NRS (mean 28 kPa; standard deviation (SD) = 14 kPa) but not size (mean 9.5 *μ*m; SD = 0.94 *μ*m), suggesting they did indeed exist as a heterogeneous population with regard to their mechanical properties (Supplementary [Supplementary-material supplementary-material-1]).

### 2.4. Intravital Microscopy to Assess HSC Homing *In Vivo* to the IR Injured Gut

Harvested HSCs (from Device C) were PBS washed and then resuspended to fluorescently label them in 4 ml PBS containing 5 *μ*M carboxyfluorescein diacetate succinimidyl ester (CFDA-SE; 10 min). Cells were then centrifuged and resuspended in 200 *μ*l warm StemPro media (37°C) prior to injection in anaesthetised male C57BL/6 mice (8–12-week-old; *N* = 5/group; Harlan, UK). All experiments were performed in accordance with the Animals Act of 1986 (Scientific Procedures; PPL:7008204 held by Dr. Kalia). Small intestinal ischaemia-reperfusion (IR) injury was induced by occluding the superior mesenteric artery for 45 minutes and then reperfusing the gut after clamp removal. The intestinal mucosal surface, the region most susceptible to IR injury, was exposed for intravital imaging as previously described [25], and the mucosal villi were visualised using a motorised inverted Olympus IX-81 microscope (Olympus, UK). A single field of view was randomly selected prior to cell infusion and imaged using a ×10 objective. A bolus dose of 2 × 10^6^ HSCs was injected via a cannulated carotid artery at 30 minutes postreperfusion. Digital videos were continuously recorded for one minute every 5 minutes and for an hour postreperfusion. Numbers of freely flowing and firmly adherent cells per field of view at each time point were counted.

### 2.5. Statistical Analysis

Values for the mechanical property parameters of the HSCs are presented as mean ± SD. The paired Student *t*-tests were performed to determine significant differences among the mechanical properties of different samples, with statistical significance considered when *p* < 0.05. Each experiments were repeated at least 3 times. For intravital experiments, *n* = 5 mice were used in each group with statistical comparisons made by two-way ANOVA, followed by Sidak post hoc tests for individual time points. All data are again presented as mean ± SD with statistical significance considered when *p* < 0.05. All statistical analyses were performed using GraphPad Software (GraphPad Software Inc., USA).

## 3. Results

### 3.1. Performance of the Two Straight Microchannel Devices at Varying Flow Rates

#### 3.1.1. Device A

**Figure 1:**
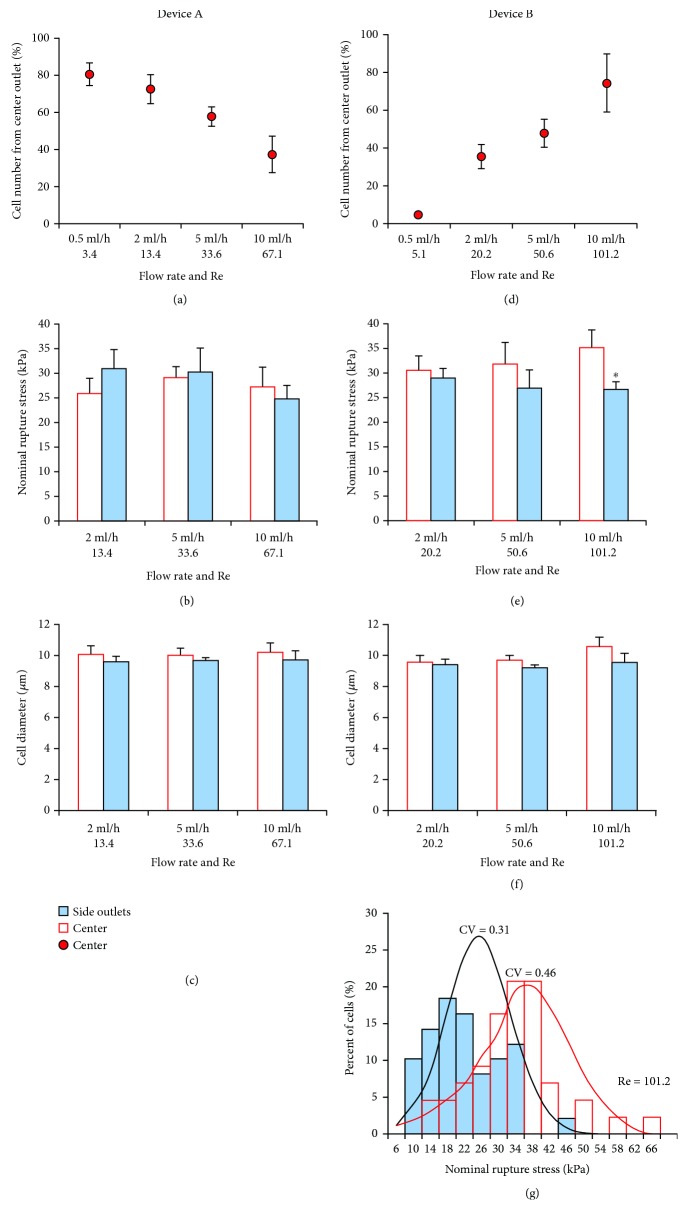
Separation efficiency of HSCs using straight Devices A and B. (a) In Device A, increased flow rate/Re directed cells away from the center outlet to the outer side outlets. (b) No significant difference in NRS (*σ*_R_), as determined using micromanipulation, between subpopulations collected from center and side outlets. (c) Size of cells in subpopulations was also similar. (d) In Device B, increased flow rate directed cells to the center outlet. (e) Cells in the center outlet had higher NRS. Separation efficiency increased with increases in flow rate. (f) Size of cells in subpopulations was similar. (g) Percentage of cells collected from the center and side outlets at a flow rate of 10 ml/h plotted against a distribution range for NRS. HSCs collected from side outlets had a narrower distribution (CV = 0.31) than cells collected from center outlet (CV = 0.46), suggesting cells in the inner outlet were mostly deformable cells. HSCs collected from the center outlet covered a wider spread of NRS with both deformable stiffer cells collected. ^∗^*p* < 0.05 as determined using a paired Student *t*-test.

As flow rate (and thus Re) increased, cells migrated towards the outer side outlets with less cells collected from the center outlet (Figure 1(a)). When flow rate was low (0.5 ml/h), approximately 80% of cells focused near the channel center indicating cells were barely separated at this flow rate. At intermediate flow rates (2 ml/h, 5 ml/h), better separation was observed. When flow rate was the highest (10 ml/h), approximately 70% of cells reached the side outlets, again indicating poor separation. Since effective cell separation with a high throughput was required, the lowest flow rate was not tested in micromanipulation experiments. For the other flow rates, no significant difference in NRS or size between cells collected from center and side outlets was observed (Figures 1(b) and 1(c)).

#### 3.1.2. Device B

In contrast to Device A, as flow rate increased, cells migrated to the center outlet (Figure 1(d)). Again, the lowest flow rate (0.5 ml/h) demonstrated very poor separation. At the highest flow rate (10 ml/h), cells collected from the side outlets had significantly (*p* < 0.05) lower NRS values, indicating they were more deformable/less stiff than those collected in center outlets (Figure 1(e)). No significant difference in cell diameter was observed at any flow rate (Figure 1(f)). The percentage of cells collected from center and outer side outlets at 10 ml/h was further plotted against a distribution range for NRS. HSCs collected from side outlets had a lower mean NRS (26.81 kPa) and narrower distribution (CV = 0.31) than cells collected from the center outlet (mean NRS = 35.16 kPa; CV = 0.46), suggesting side outlets collected mostly deformable cells (Figure 1(g)). However, the center outlet cells had a widespread of NRS values with both deformable and stiff cells collected. Cell throughput at this flow rate was 2.3 × 10^5^ cells/min.

### 3.2. Performance of the Three Spiral Microchannel Devices at Varying Flow Rates

**Figure 2:**
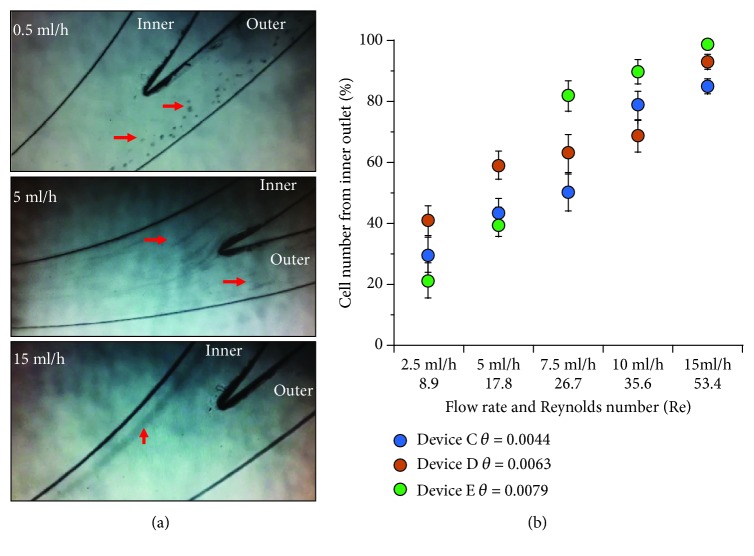
Performance of the three spiral microchannel devices at varying flow rates. (a) When imaged microscopically, it was noted that HSCs moved to outer, inner/outer, or inner outlets at low (0.5 ml/h), intermediate (5 ml/h), or high (15 ml/h) flow rates in Device C. It is not possible to see individual cells at the higher flow rates although streaks left by fast moving cells can be seen. (b) For all three devices, as flow rate/Re increased, HSCs gradually shifted to the inner outlet.

For all three spiral devices, changing the flow rate focused HSCs to different lateral positions (Supplemental online videos [Supplementary-material supplementary-material-1]). As flow rate/Re increased, the percentage of HSCs collected from the inner outlet increased (Figures 2(a) and 2(b)). Since a loss of separation was noted at low (0.5 ml/h, 2.5 ml/h) and high (15 ml/h) flow rates, only intermediate flow rates (5 ml/h, 7.5 ml/h, and 10 ml/h) were chosen for further mechanical testing of harvested cells. Low flow rates were also associated with cell sedimentation in the syringe. For Devices D and E, >80% of cells migrated near the inner side wall at 10 ml/h resulting in a low separation. Therefore, in these two devices, only flow rates of 5 ml/h and 7.5 ml/h were further used.

#### 3.2.1. Device C

**Figure 3:**
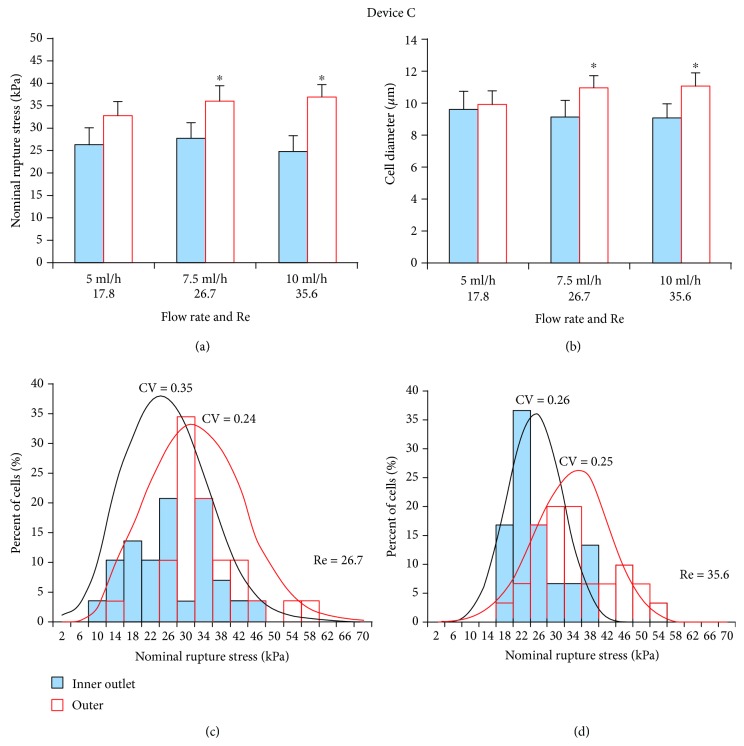
Separation efficiency of HSCs using spiral Device C. (a) At high flow rates (7.5 ml/h and 10 ml/h)/Re, inner outlet HSCs had significantly lower NRS values and were thus more deformable than outer outlet HSCs. (b) Mean diameter of inner outlet HSCs was significantly smaller for the two higher flow rates. (c) At the lower flow rate (7.5 ml/h; CV = 0.35), NRS distribution of inner outlet HSCs was wider than at the (d) higher flow rate (10 ml/h; CV = 0.26). Hence, this device performed more sensitive sorting at 10 ml/h. ^∗^*p* < 0.05 as determined using a paired Student *t*-test.

At both 7.5 ml/h and 10 ml/h flow rates, cells collected from inner outlets had significantly (*p* < 0.05) lower NRS values, indicating they were more deformable. They were also significantly (*p* < 0.05) smaller than those collected from outer outlets (Figures 3(a) and 3(b)). NRS distribution for these two flow rates indicated that the mechanical strength of cells collected from the inner outlet was more dispersed at 7.5 ml/h (CV = 0.35; Figure 3(c)) than at 10 ml/h (CV = 0.26; Figure 3(d)). Therefore, this device performed more sensitive sorting at 10 ml/h. Cell throughput at this flow rate was 2.3 × 10^5^ cells/min.

#### 3.2.2. Device D

**Figure 4:**
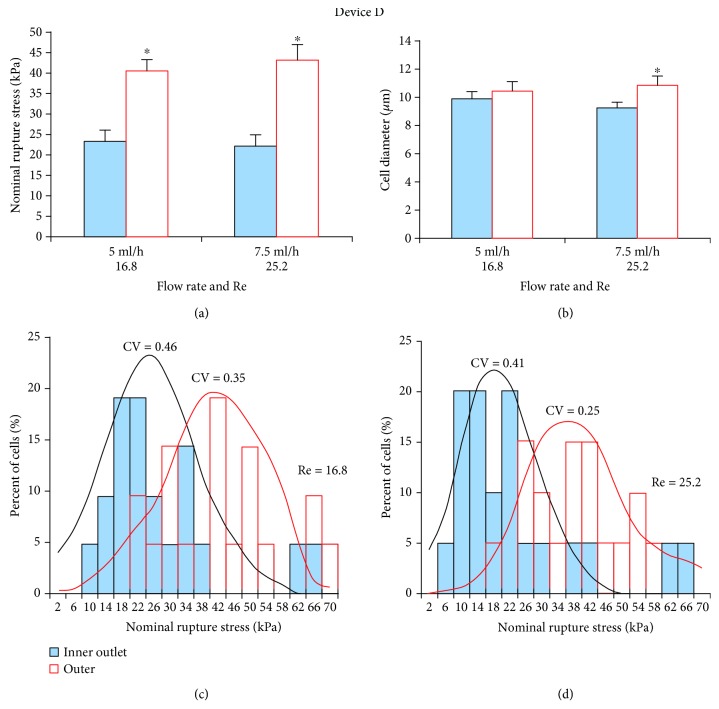
Separation efficiency of HSCs using spiral Device D. (a) At both flow rates (5 ml/h and 7.5 ml/h), inner outlet HSCs had significantly lower NRS values and were thus more deformable than outer outlet HSCs. (b) Mean diameter of inner outlet HSCs was significantly smaller only at the higher flow rate. (c) At the lower flow rate (5 ml/h), NRS distribution of inner outlet HSCs was wider (CV = 0.49) than at the (d) higher flow rate (10 ml/h; CV = 0.41). Hence, this device performed more sensitive sorting at 7.5 ml/h. ^∗^*p* < 0.05 as determined using a paired Student *t*-test.

At both 5 ml/h and 7.5 ml/h flow rates, cells collected from inner outlets had significantly (*p* < 0.05) lower NRS values but were only significantly (*p* < 0.05) smaller in size at 7.5 ml/h (Figures 4(a) and 4(b)). NRS distribution for these two flow rates indicated that the mechanical strength of cells from the inner outlet was more dispersed at 5 ml/h (CV = 0.49; Figure 4(c)) than at 7.5 ml/h (CV = 0.41; Figure 4(d)). Therefore, this device performed more sensitive sorting at 7.5 ml/h. Cell throughput at this flow rate was 1.5 × 10^5^ cells/min.

#### 3.2.3. Device E

**Figure 5:**
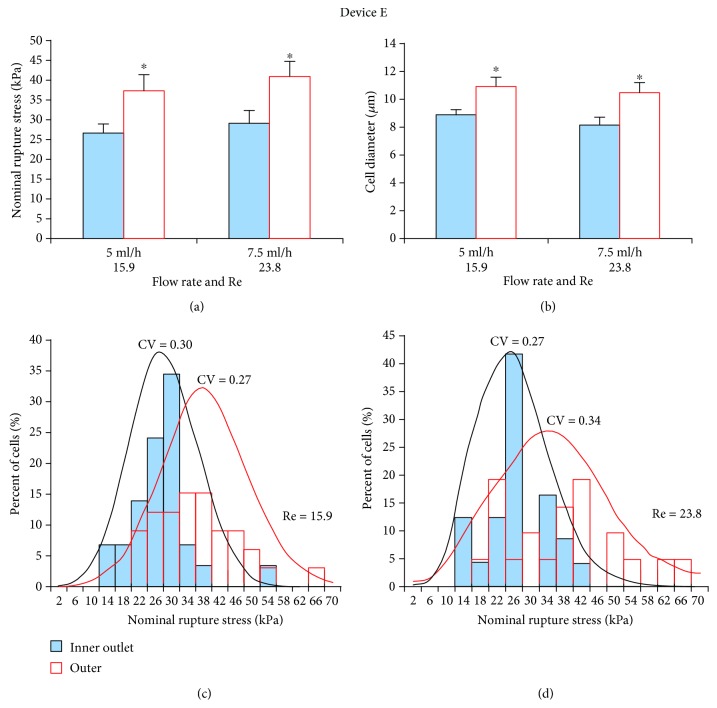
Separation efficiency of HSCs using spiral Device E. (a) At both flow rates (5 ml/h and 10 ml/h), inner outlet HSCs had significantly lower NRS values and were thus more deformable than outer outlet HSCs. (b) Mean diameter of inner outlet HSCs was significantly smaller for both flow rates. (c) At the lower flow rate (5 ml/h), NRS distribution of inner outlet HSCs was wider (CV = 0.30) than at the (d) higher flow rate (10 ml/h; CV = 0.27). Hence, this device performed more sensitive sorting at 7.5 ml/h. ^∗^*p* < 0.05 as determined using a paired Student *t*-test.

At both 5 ml/h and 7.5 ml/h flow rates, cells collected from inner outlets had significantly (*p* < 0.05) lower NRS values and were also significantly (*p* < 0.05) smaller than those collected from outer outlets (Figures 5(a) and 5(b)). NRS distribution indicated that the mechanical strength of cells from the inner outlet was more dispersed at 5 ml/h (CV = 0.30; Figure 5(c)) than at 7.5 ml/h (CV = 0.27; Figure 5(d)). Therefore, this device also performed more sensitive sorting at 7.5 ml/h. Cell throughput at this flow rate was 1.6 × 10^5^ cells/min.

Overall, Device C focused more deformable and smaller cells into the inner outlet at a flow rate of 10 ml/h, with a relatively higher separation efficiency (lowest CV value of 0.26) and higher throughput than the other devices. Hence, this device was further utilized for intravital experiments at a flow rate of 10 ml/h. The performance of the 5 different microfluidic systems is summarized in Supplementary [Supplementary-material supplementary-material-1].

### 3.3. Relationship between NRS and Cell Size before and after Separation with Device C

**Figure 6:**
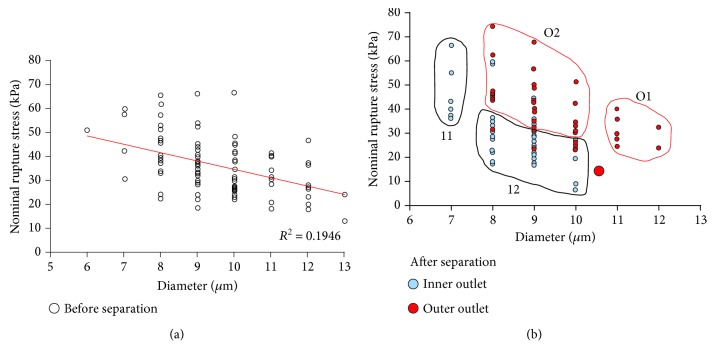
Relationship between NRS and size for HSC populations before and after separation using Device C. (a) Correlative relationship between NRS and HSC diameter before separation. NRS is weakly and negatively correlated with diameter. *N* = 100. (b) Correlative relationship between NRS of HSCs collected from inner and outer outlets. Four distribution patterns were identified with cells harvested from the outer outlet located mostly at the right (O1) and top (O2) of the distribution graph and cells harvested from the inner outlet located mostly at the left (I1) and bottom (I2). The numbers 1 and 2 simply denote whether the cells are on the far ends of the diameter range (I1 and O1) or in the midrange (I2 and O2). *N* = 50.

Prior to separation, NRS was weakly but negatively correlated with cell diameter (correlation coefficient (*R*^2^) = 0.1946; Figure 6(a)). In cells separated using Device C, four distribution patterns were identified with cells harvested from the outer outlet located mostly at the right (O1) and top (O2) of the distribution graph and cells harvested from the inner outlet located mostly at the left (I1) and bottom (I2) (Figure 6(b)). Cells with a significant size difference (i.e., those in the left-most and right-most of the size distribution graph) migrated in opposite directions with smaller cells focusing to the inner channel walls (I1) and larger cells in the outer channel walls (O1) regardless of their deformability. In this case, size may play a dominant role in cell migration. However, most of the cells were of similar size, i.e., between 8 and 10 *μ*m, and were separated primarily based on their mechanical strength variation. This was responsible for the smaller NRS value for cells collected in the inner channel (I2) compared to the higher value for cells collected in the outer channel (O2).

### 3.4. Intravital Microscopy to Assess the Trafficking of HSCs Isolated Using Device C

**Figure 7:**
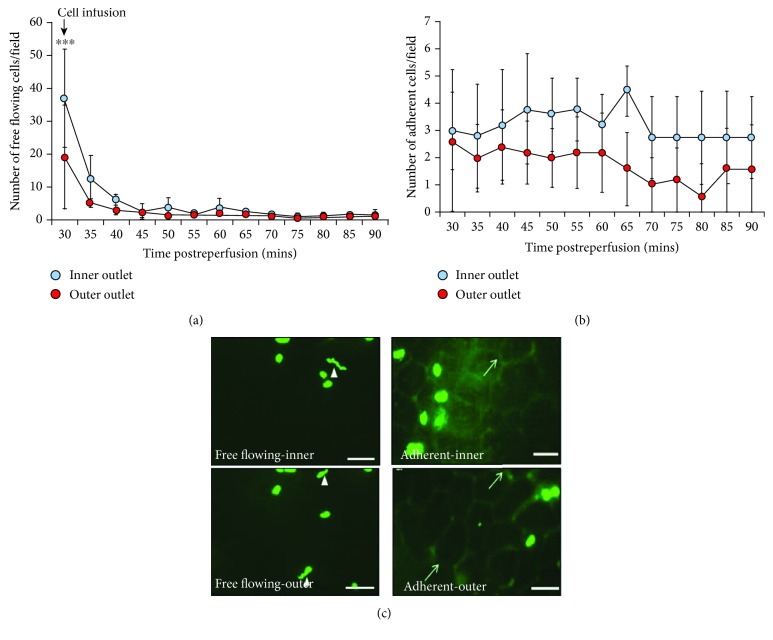
Free-flowing and adherent HSCs, separated using Device C, in IR injured mouse small intestine. (a) Only at the point of infusion (30 mins postreperfusion) were significantly more inner outlet/deformable HSCs observed to pass through the IR injured gut mucosa compared to outer outlet/stiffer cells. (b) No significant difference in HSC adhesion was observed. (c) Intravital images of freely flowing and adherent CFSE-labelled inner outlet and outer outlet HSCs in the mucosal microcirculation. Velocity of some circulating cells was very high and resulted in them appearing as streaks (arrowheads). Although numbers look similar in static images, over a continuous recording period of 1 minute, more circulating cells would have been quantitated. In some images, the outline of mucosal villous capillaries can be seen (arrows). Results are presented as mean ± SD. *N* = 5. ^∗∗∗^*p* < 0.001. Scale bar = 50 *μ*m.

Trypan blue was used to verify the viability of HSCs after they had passed through Device C at 10 ml/h. No significant difference was observed in the number of viable cells passing through the microchannels when compared to cells which did not undergo separation (Supplementary [Supplementary-material supplementary-material-1]). The number of Device C separated HSCs freely flowing through the gut was the highest at the point of infusion (i.e., at 30 mins postreperfusion) for all cells, regardless of collection channel, and then rapidly decreased at later observation times (Figure 7(a)). However, for at least 10 minutes postinfusion (i.e., between 30 and 40 mins postreperfusion), more free-flowing inner outlet/more deformable HSCs were observed passing through the mucosal microcirculation than outer outlet/stiffer cells. This was only significant (*p* < 0.001) though immediately after infusion of cells. Indeed, the number of free-flowing deformable cells counted at 30 minutes postreperfusion was double the number of free-flowing stiffer cells (Figure 7(b)). This was an underestimation of the actual number of trafficking cells as many had a velocity too high to allow them to be counted. This difference decreased with time until no significant difference was present between cells collected from the two outlets at the end of the observation period. However, at all time points, HSCs could still be observed trafficking through the injured gut. There was a general trend to observe more HSCs harvested from the inner outlet adherent within the mucosal microcirculation. However, this did not attain statistical significance (Figures 7(b) and 7(c)).

## 4. Discussion

To attain the most efficient and high-throughput separation of HSCs based on their mechanical properties, 2 straight and 3 spiral microfluidic devices with different geometric designs were fabricated. This allowed the effects of changes in aspect ratio (AR) and Reynolds number (Re) and the addition of a curvature ratio (*θ*) to be investigated. We firstly tested whether a significant variation in the mechanical strength of HSCs actually existed in a single cell population which could be exploited for separation purposes. A narrow variation in size (6-12 *μ*m; SD = 0.94 *μ*m) was identified which meant sensitivity for isolation purposes was likely not high. However, a wide variation in NRS (2-64 kPa; SD = 14 kPa), an indicator of cell deformability/stiffness, was demonstrated which fitted a normal Gaussian distribution. This meant this mechanical property could indeed be exploited for sorting a heterogeneous population of HSCs in microfluidic devices. This broad range also clearly underscored the need to identify microfluidic isolation methods that can enrich for a more homogeneous subpopulation of more deformable cells. In the current study, spiral microfluidic Device C provided the best performance in terms of generating subpopulations of cells with the most significant differences in both cell size and NRS. Moreover, intravital studies showed that the sorted cell subpopulation of lower rupture force resulted in more free-flowing cells passing through the injured gut in a short period immediately upon infusion. Interestingly, this did not result in a significant increase in HSC adhesion to mucosal villous microvessels.

Two straight rectangular microchannels were tested. Generally, rectangular microchannels are more widely employed in cell focusing and separation methods than square or circular channels as they appear to more effectively separate particles [29]. Results demonstrated that the focusing of cells in straight microchannels behaved differently and in a complex manner. The differential cell focusing behaviors in straight channels are explained by considering two competing “lift forces” (*F*_L_) acting on the cells perpendicular to the direction of Poiseuille flow (Supplementary [Supplementary-material supplementary-material-1]a). These are the shear-gradient lift force (*F*_LS_) directing cells to the channel walls and the wall-induced lift force (*F*_LW_) moving cells to the direction of the channel centerline. It is accepted that both forces increase with increasing flow rate or Re [29–33]. In Device A which had the smaller aspect ratio (AR = 5), an increase in flow rate likely increased *F*_LS_ more so than *F*_LW_. As *F*_LS_ becomes more dominant, it consequently leads to more cells moving towards the channel wall. This was indeed observed as the number of cells being collected from the center outlet decreased with increased flow rate. Device B was wider, but height was reduced leading to a larger aspect ratio (AR = 10). Therefore, the velocity profile became flattened and likely increased *F*_LW_ above the increase of *F*_LS_. Consequently, it was observed in this device that more cells migrated to the channel centerline as flow rate increased. The net *F*_L_ is also proportional to the cell size. For bigger cells, the magnitude of net *F*_L_ grows faster with the flow rate. Therefore, smaller cells move more slowly than bigger ones and become entrained near the centerline of device A and towards the side walls of device B. Interestingly, no difference in the size of subpopulations was observed with either Device A or Device B which may be due to the small variation in HSC size.

Cells are not solid or rigid structures but are deformable. This deformability will induce additional lift forces. Similar to *F*_LS_ and *F*_LW_, deformability-induced lift force (*F*_LDeformation_) also acts perpendicular to flow [34]. Therefore, theoretically for cells with similar diameter, the more deformable HSCs could be displaced by *F*_LDeformation_ away from the migration direction of relatively rigid cells, resulting in them being separated. However, since no effective mechanical separation was observed with Device A, it is possible that the channel length was not sufficiently long enough to enable cell separation. In Device B, more deformable cells were displaced towards the channel walls and thus collected from side outlets at high flow rates and Re. Moreover, due to the greater difference between the changes of the two lift forces, the channel length was long enough for cells to be significantly separated.

For curved channels, similar trends in cell number and NRS distribution were observed in the three channels with different curvature ratios. Though the exact mechanism responsible for cell sorting in curved channels is still unclear and requires further investigations, some hypothesis based on experiments and simulation have been proposed and could help improve our understanding of the cell migration [34, 35]. When fluid flows through a curved channel, a secondary flow called Dean drag, centrifugal, or vortex flow (*F*_D_) arises which is characterised by two counter-rotating vortices in the height direction and perpendicular to the primary flow direction (Supplementary [Supplementary-material supplementary-material-1]b). As such, flow is directed outwards near the channel center and inwards near the top and bottom walls [36]. In addition to adding Dean drag force, the curvature can also change *F*_LS_ through redistributing the velocity profile which can change the vertical position of cells. This redistribution becomes significant with the increase of the curvature ratio (*θ*) value [37].

Based on this hypothesis, the cell migration in one spiral channel at varying flow rate is discussed here, and the other two follows a similar pattern. The *F*_LS_ in the vertical direction in the inner half of the channel decreases compared to the parabolic profile in the *θ* = 0 case, and *F*_LW_ or wall-induced inertial becomes more dominant which allows cells to take vertical equilibrium positions near the channel center [38]. By crossing the vertical position, the bigger and/or rigid cells move faster towards the center and experience a switch in the direction of Dean flow, moving outwards in the channel. Smaller and/or more deformable cells near the top or bottom walls move inwards; thus, separation can be achieved. Indeed, this was observed for all three spiral devices whereby smaller cells with lower NRS (more deformable) were harvested from the inner outlets and larger cells with larger NRS (more rigid) were collected from the outer outlets. With the increasing Re, the shear gradient became higher [17], which is enough to counter *F*_D_, and so, more cells tend to move away from the channel center and are directed towards the inner half. Eventually, all the cells were dragged inwards. This proposed hypothesis can explain the behaviors of cell migration with the increase of flow rate in each spiral device.

Previous *in vivo* studies have reported that the systemic infusion of stem cells did not yield many cells reaching the organ of interest primarily due to the majority of cells being trapped in remote capillaries [5–8]. Therefore, it was hypothesized that after systemic injection, the smaller and more deformable HSCs harvested from Device C would avoid nonspecific entrapment and thus lead to increased numbers in the peripheral circulation. Intravital studies clearly demonstrated that double the number of inner outlet cells of Device C trafficked through the imaged mucosal surface in a one-minute period of continuous recording immediately upon infusion when compared to stiffer outer outlet cells. The blood circulation time for a mouse is approximately 4-6 seconds [8, 39] which means the blood circulates about 10 times during the 1-minute visualisation period. Hence, injected HSCs would have passed around the body/lungs/gut etc. approximately 10 times during this period of recording. These findings demonstrate that HSCs isolated from the inner outlet do have advantages over those from the outer outlet due to their smaller size and lesser stiffness, which permits their better retention within the peripheral blood. However, this advantage appears to have lasted for about 10 minutes. Thereafter, the majority of cells from both groups were lost from the peripheral circulation and appeared entrapped in the lungs as shown by us previously [40].

Small microspheres (4-5 *μ*m) can pass straight through the lungs while the majority of larger SCs such as mesenchymal stromal cells (MSCs) (15-19 *μ*m) are trapped in the pulmonary system [5]. Similar observations were made by Fischer and colleagues who demonstrated that the passage of bone marrow-derived mononuclear cells (7 *μ*m) was 30-fold higher compared to MSCs (18 *μ*m) [4]. These significant differences in lung entrapment between MSCs and other blood cells are likely due to the considerable difference in their sizes. However, for HSCs, the mean diameter of Device C-isolated cells from both outlets was 9.1 ± 0.5 *μ*m and 10.5 ± 0.4 *μ*m. It is not anticipated that this degree of difference in size would be enough to result in a significant difference in the number of cells in circulation. Therefore, it is highly likely that the NRS/deformability differences between inner and outer outlet cells contributed to the increase in the number of free-flowing cells in first minute observation. These novel results clearly highlight the critical impact HSC rigidity/softness has on their trafficking capabilities to injured tissues.

Although increased homing to and trafficking through the injured gut was observed, disappointingly, this did not result in enhanced adhesion and thus intestinal retention. This may be due to the cells not becoming sufficiently activated to adhere while transiting through the gut. Firm HSC-endothelial interactions are regulated by the interplay of a whole host of soluble inflammatory factors including cytokines and chemokines that activate both cell types to express adhesion molecules [8, 9]. This adhesion cascade is very similar to that observed for inflammatory leukocytes. Recent evidence by our group and others has shown that modulating the expression of adhesion molecules and/or activator chemokines is able to enhance HSC adhesion within injured organs and also to the bone marrow [8, 40–42]. For example, we have previously shown that HSC pretreatment with chemokines such as CXCL12 (SDF-1*α*) or KC (murine functional IL-8 homologue) significantly increased HSC adhesion within ischaemically injured kidney, while SDF-1*α* also increased numbers continuing to circulate in the peripheral blood [8]. Also, pretreatment with the free radical hydrogen peroxide significantly improved HSC retention with the ischaemic and colitic gut [40, 41]. We therefore postulate that increasing HSC trafficking using microfluidic systems, combined with cell pretreatment strategies to preactivate or overexpress surface adhesion molecules, would be an effective dual strategy to enhance the efficiency of SC therapy. This remains to be validated in future work.

Whether the subsequent use of the smaller/deformable HSC subpopulations affects their therapeutic ability will also require further investigation. Interestingly, Wagner and colleagues separated human CD34^+^ cells using counterflow centrifugal elutriation into three different sizes and found that the smallest cells (<9.5 *μ*m) were most functionally effective as determined by their ability to continuously proliferate [43]. However, all sizes could repopulate the foetal thymus/liver in SCID mice. Interestingly, the smallest cells highly expressed receptors for multiple inflammatory factors including IL-1, IL-6, G-CSF, SCF, and MIP-1*α*. This is important as it likely aids in HSC activation by these agonists, found in inflamed tissues, allowing subsequent release of therapeutic factors. More recent investigations have also demonstrated that smaller diameter and less stiff subpopulations of culture-expanded stromal cells from both adult and foetal bone marrow are highly clonogenic and also exhibit gene, protein, and functional signatures of multipotency [44]. Based on these published observations, it is possible that the smaller size of the selected HSC subpopulation may benefit rather than hinder their therapeutic efficacy.

Previous studies using microfluidic systems for cell sorting are mainly focused on separating target cell populations from heterogeneous samples of different cell types, such as isolating tumor cells from whole blood, enriching platelets from other blood cellular components, or classifying various cell types using size and deformability as markers in inertial microfluidics [45–49]. In these investigations, the mechanical differences between different cell types are generally very significant. However, the current study applied microfluidic systems to separate subpopulations from one cell type, based on the intrinsic mechanical heterogeneity of individual cells in one cell population. More recently, high-throughput inertial microfluidics has also been used to efficiently separate different subpopulations from a single heterogeneous population of bone marrow-derived MSCs [20, 44]. Indeed, larger/stiffer MSCs were identified as being committed osteoprogenitors with the smaller/compliant cells possessing multilineage potential [44]. Our study presents a novel and challenging application which can provide prospects for developing new clinical and research instruments benefiting HSC cellular therapy. Indeed, studies to understand HSC deformability [50] and utilize microfluidic devices to separate them based on deformability [51] are recently increasing and may well become a strategy to increase their therapeutic effectiveness. Importantly, our novel results indicate that fractioning HSCs by their mechanical properties is one approach to enhance their circulation time. However, future studies would need to focus on combining effective strategies that modify adhesion with those that modify homing to fully benefit cellular therapy.

## Data Availability

The raw data, including means and standard errors of mean, used to support the findings of this study are included within the article and the supplementary information files. All these raw data used to support the findings of this study are available from the corresponding authors upon request.

## Supplementary Materials

Supplementary 1 Supplementary Figure 1 presents images of straight and spiral microfluidic systems used for separating HSCs.

Supplementary 2 Supplementary Figure 2 is additional data to show that HSCs exist as heterogeneous population of cells with varying stiffness and size.

Supplementary 3 Supplementary Figure 3 is additional data to show that passing HSCs through the microfluidic devices does not cause cell death.

Supplementary 4 Supplementary Figure 4 presents images of the various forces imposed on HSCs as they pass through straight and spiral microfluidic devices.

Supplementary 5 Supplementary Table 1 presents the dimensionless numbers used in this study and equations used to derive these values.

Supplementary 6 Supplementary Table 2 presents the geometries of the 5 microfluidic devices used to separate HSCs.

Supplementary 7 Supplementary Table 3 summarizes the performance of the 5 different microfluidic systems in separating HSCs.

Supplementary 8 Supplementary Videos show HSCs being directed to different outlets of spiral microfluidic devices depending on the flow rate used.
