# Supplementary figures and images for: Designing Microfluidic Devices to Sort Haematopoietic Stem Cells Based on Their Mechanical Properties
Source: Stem Cells Int. 2019 Sep 5;2019:8540706. doi: 10.1155/2019/8540706 (PMC6748184; doi:10.1155/2019/8540706)

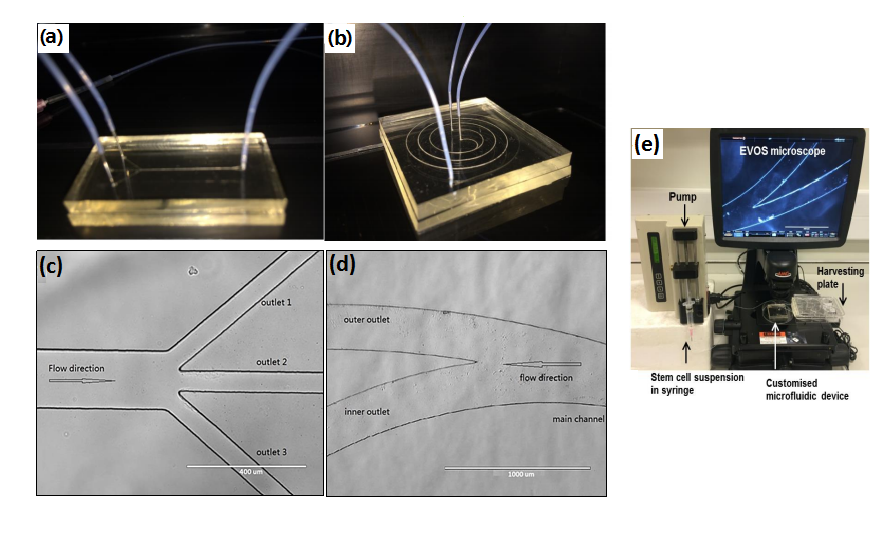

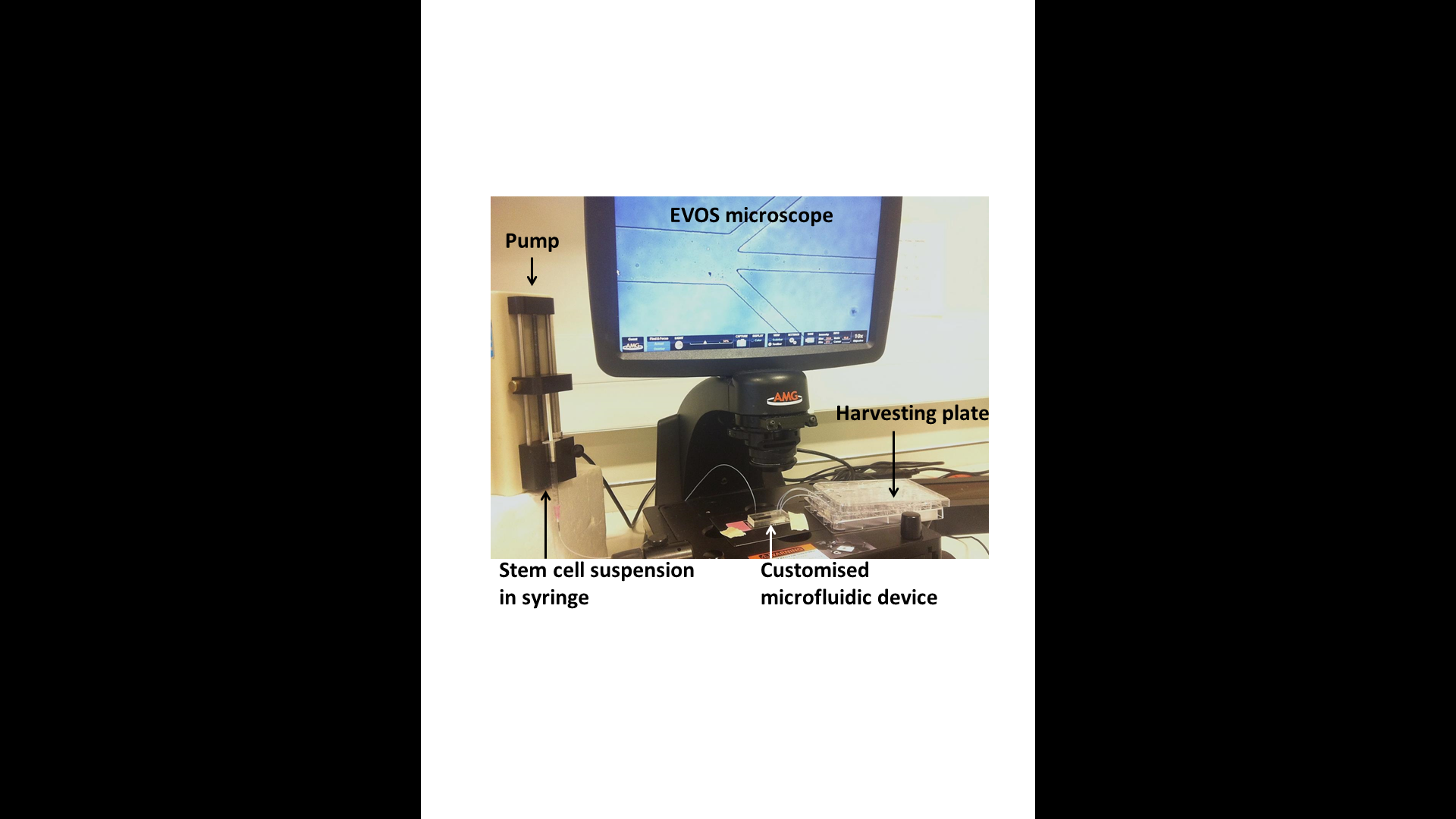

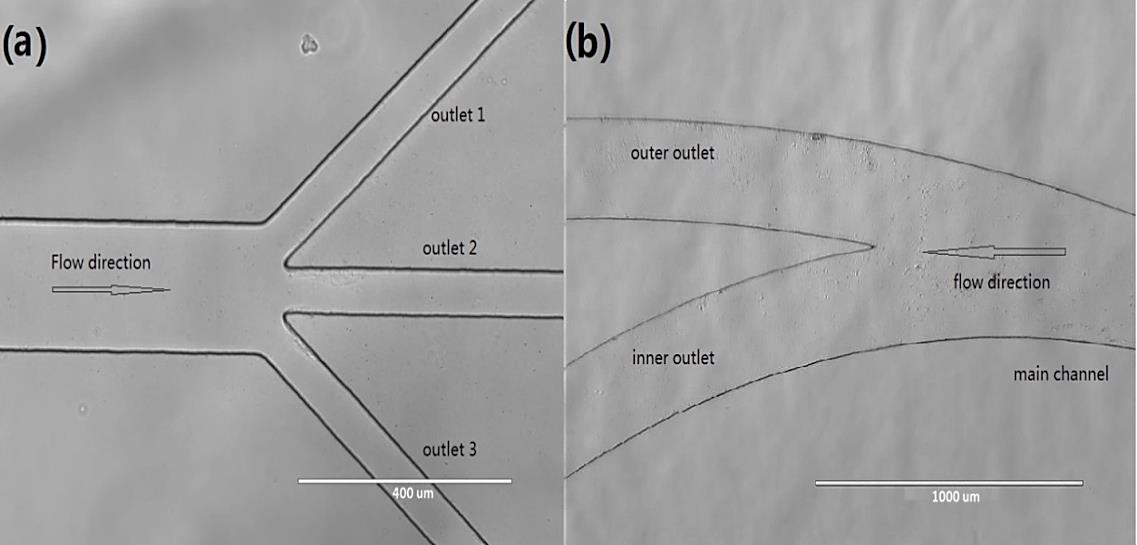

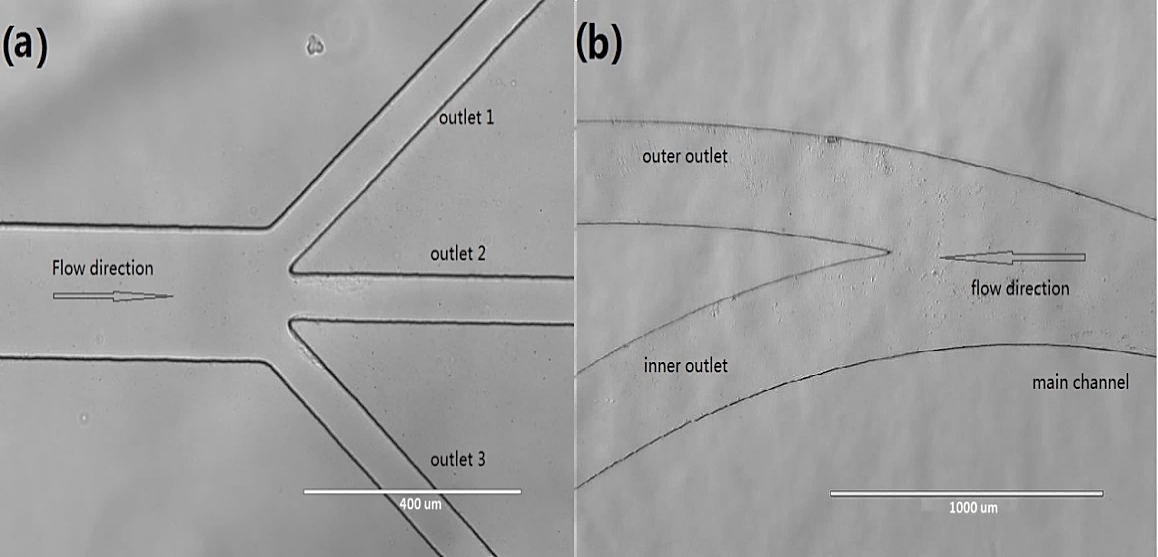

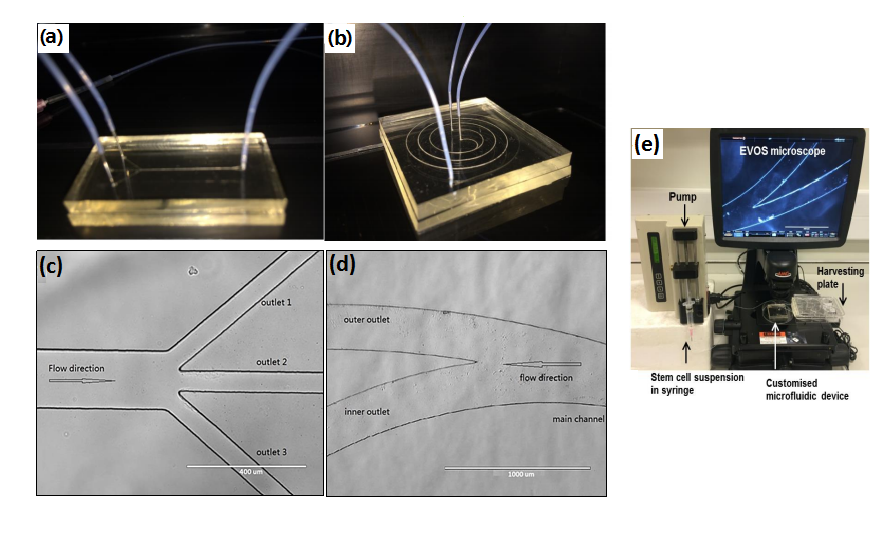


**a b**

**c d**

**e**

**Supplemental Figure 1**

Supplement: Supplementary 1 — Supplementary Figure 1 presents images of straight and spiral microfluidic systems used for separating HSCs. [file 8540706.f1.docx]

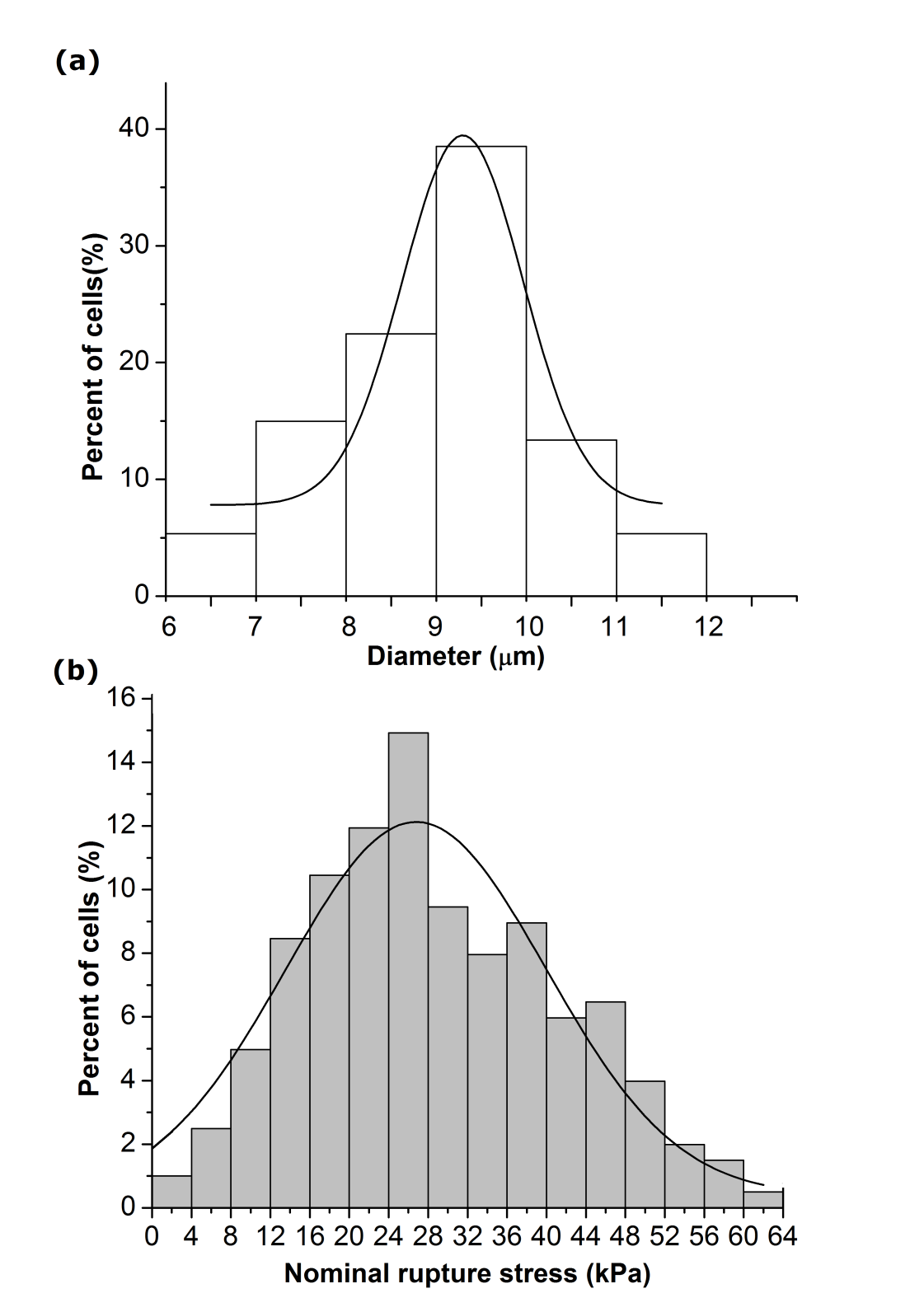


**Supplemental Figure 2**

Supplement: Supplementary 2 — Supplementary Figure 2 is additional data to show that HSCs exist as heterogeneous population of cells with varying stiffness and size. [file 8540706.f2.docx]

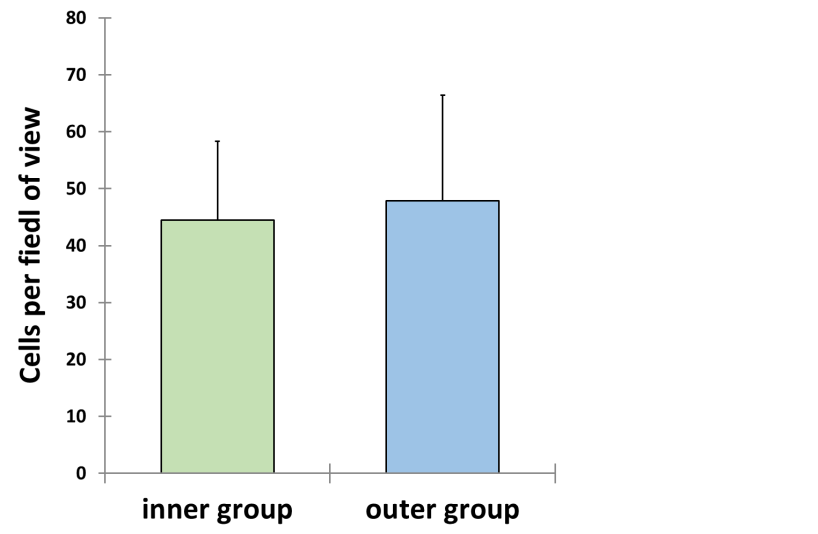

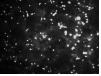

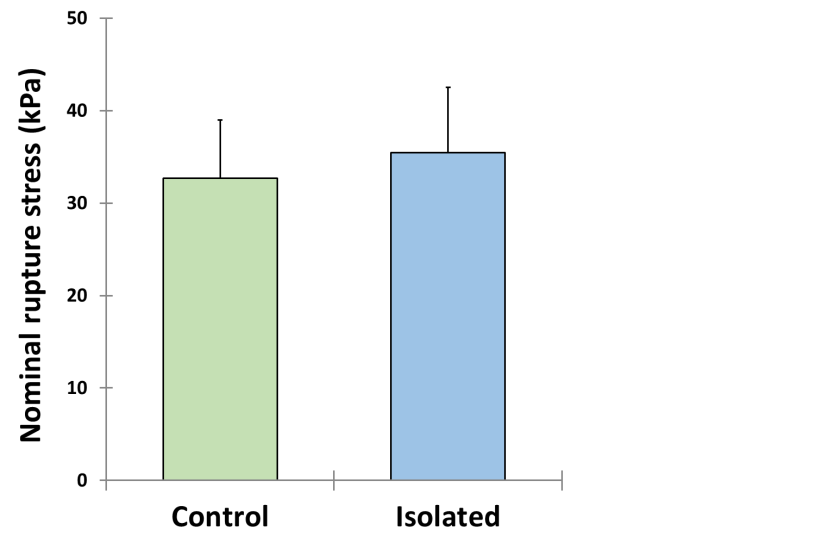

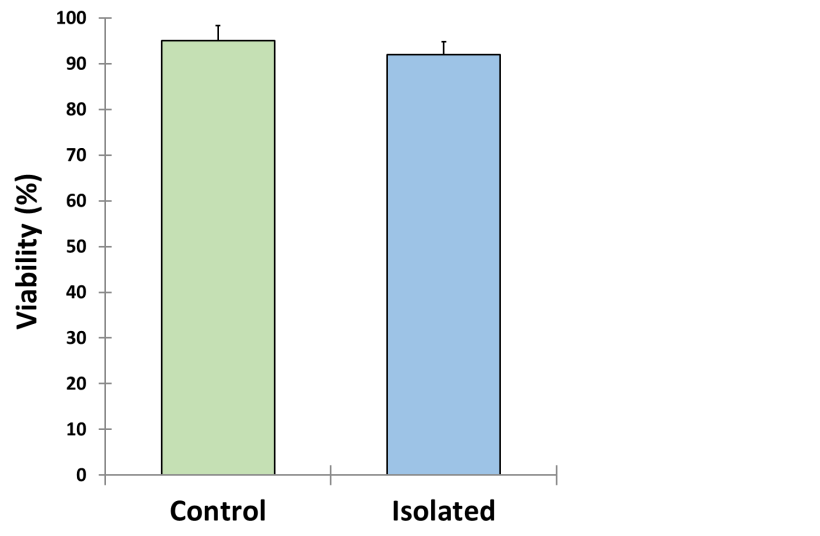


**a b**

**c d**

**Supplemental Figure 3**

Supplement: Supplementary 3 — Supplementary Figure 3 is additional data to show that passing HSCs through the microfluidic devices does not cause cell death. [file 8540706.f3.docx]

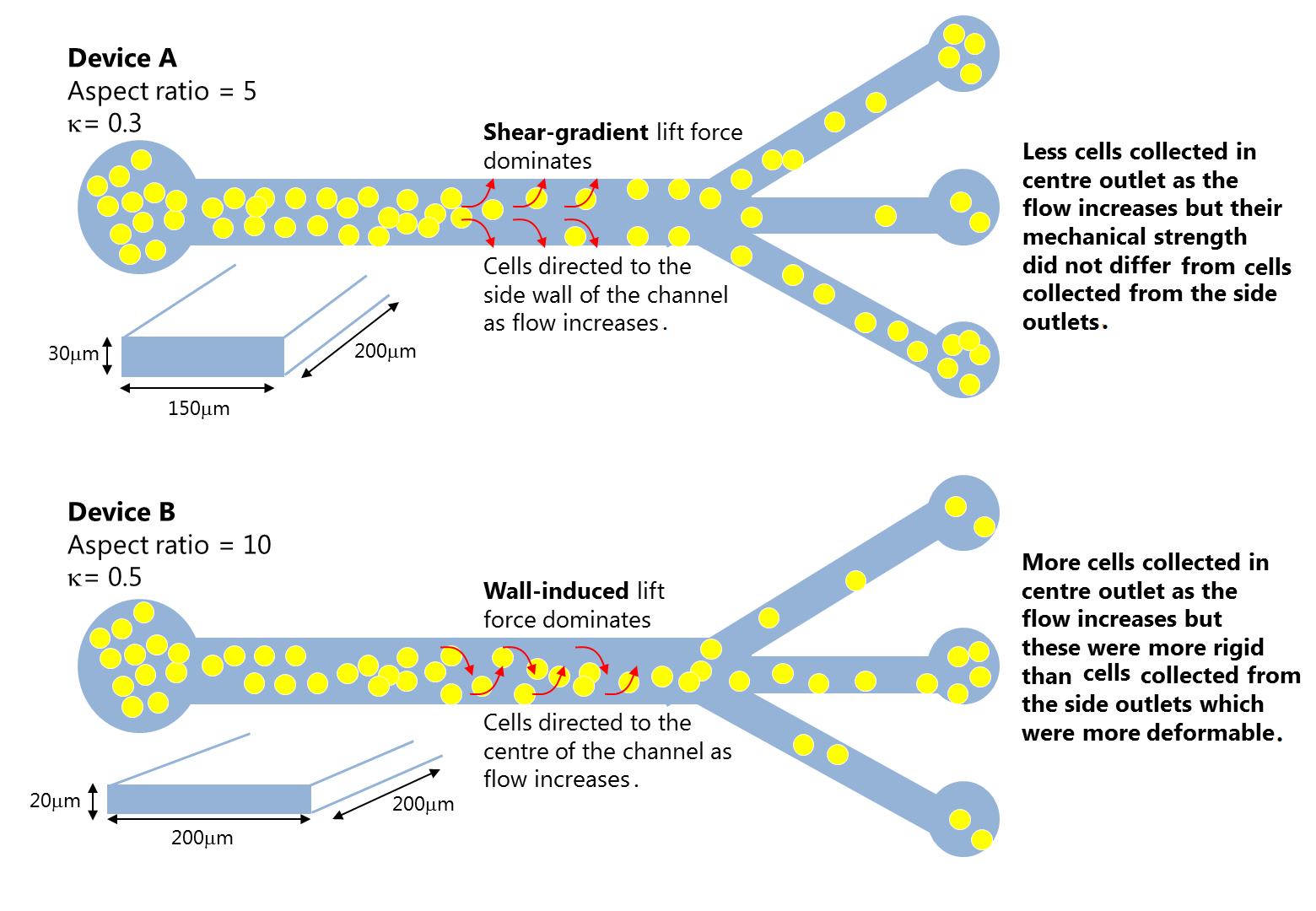

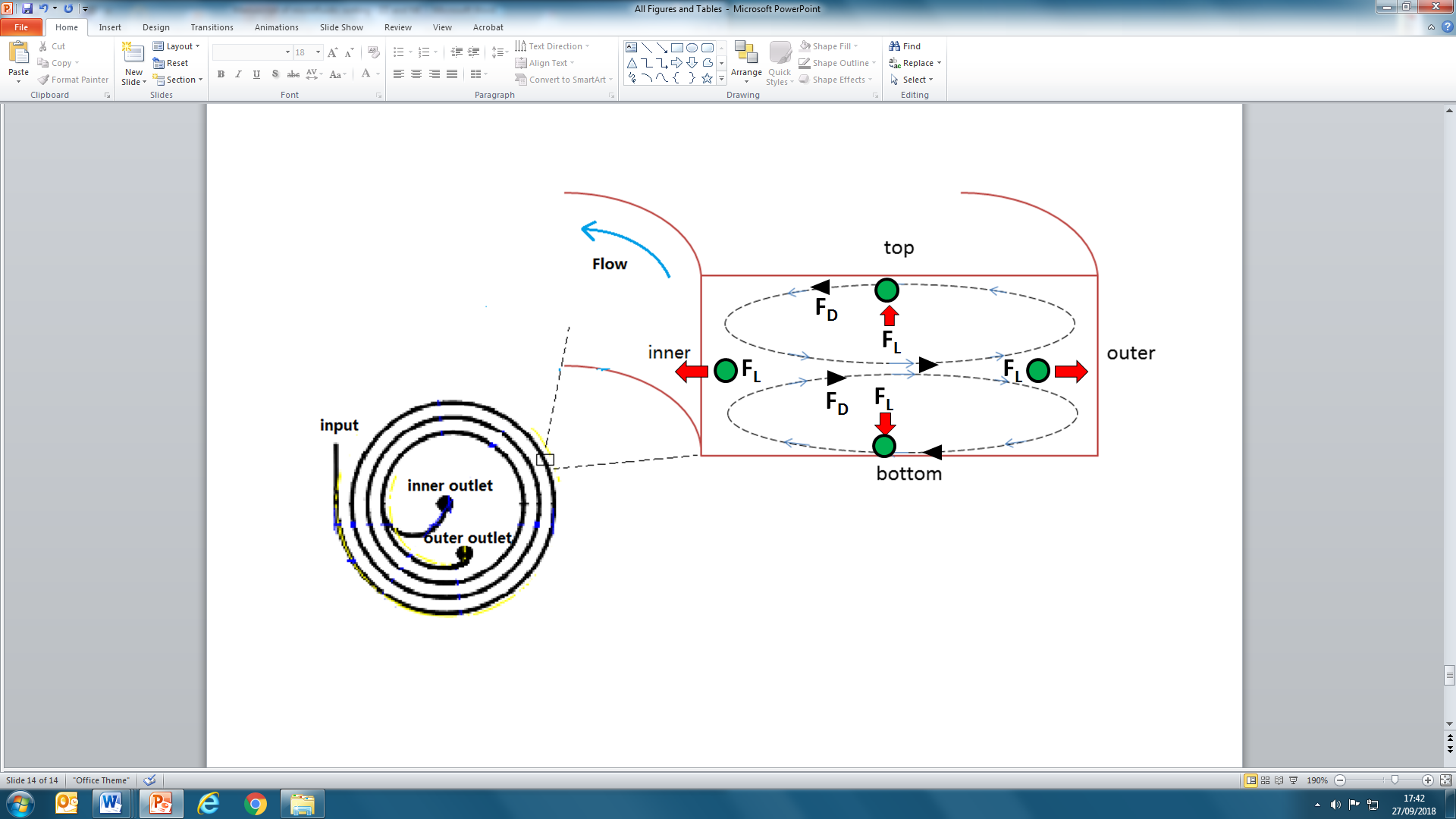


**a**

**b**

**Supplemental Figure 4**

Supplement: Supplementary 4 — Supplementary Figure 4 presents images of the various forces imposed on HSCs as they pass through straight and spiral microfluidic devices. [file 8540706.f4.docx]

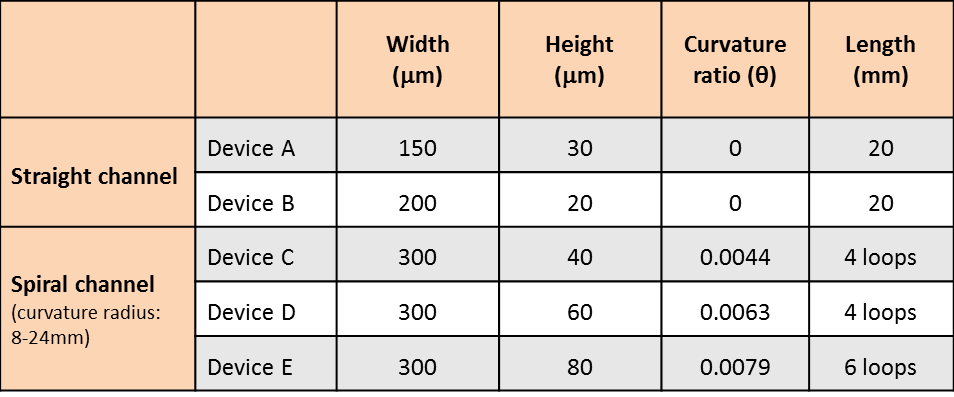


**Supplemental Table 1**

Supplement: Supplementary 5 — Supplementary Table 1 presents the dimensionless numbers used in this study and equations used to derive these values. [file 8540706.f5.docx]

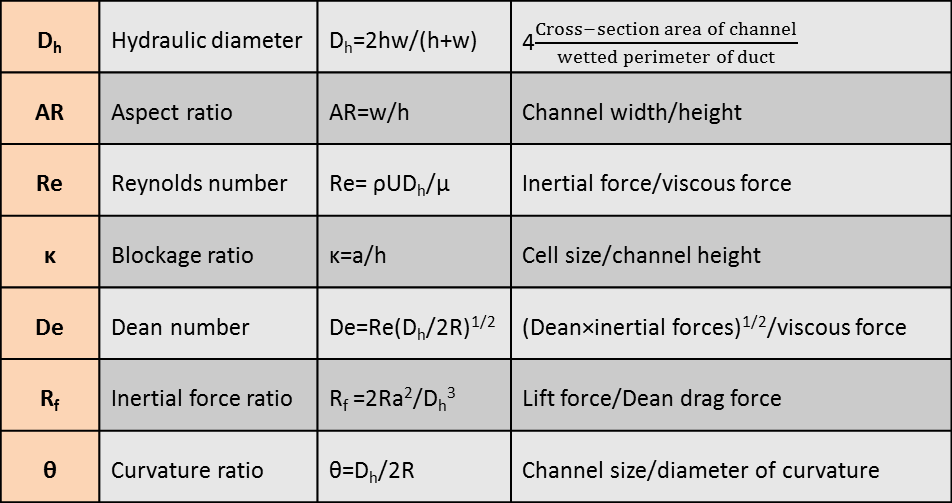


**Supplemental Table 2**

Supplement: Supplementary 6 — Supplementary Table 2 presents the geometries of the 5 microfluidic devices used to separate HSCs. [file 8540706.f6.docx]

**Supplemental Table 3**


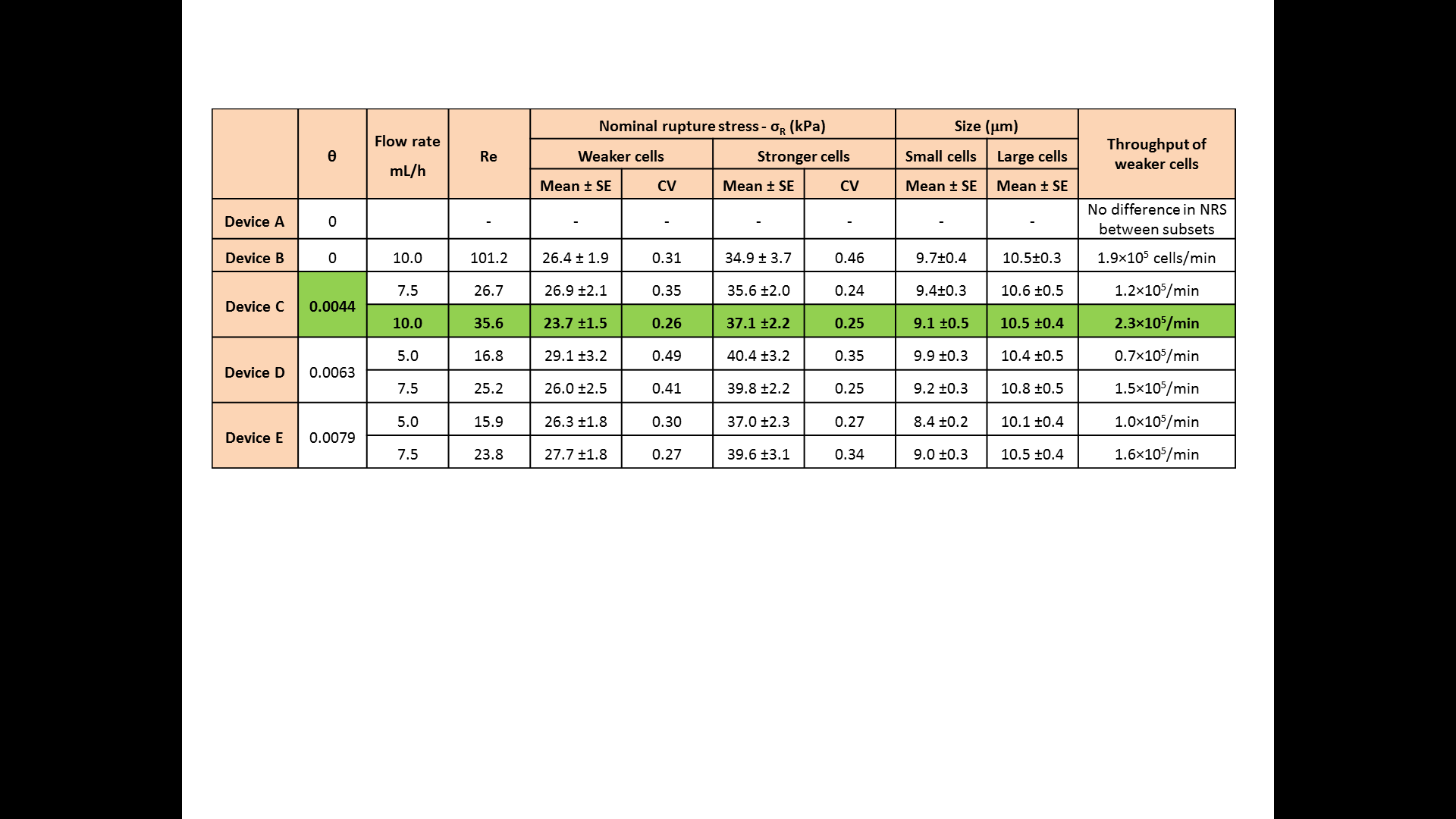

Supplement: Supplementary 7 — Supplementary Table 3 summarizes the performance of the 5 different microfluidic systems in separating HSCs. [file 8540706.f7.docx]
